# Regrouping, reforming and re-engineering: applying robotics to new challenges

**DOI:** 10.1155/S1463924696000144

**Published:** 1996

**Authors:** Stephen Scypinski, Theodore Sadlowski, John Baiano

**Affiliations:** Analytical Research and Development Hoffmann-La Roche Inc. Nutley NJ 07110 USA


Journal of Automatic Chemistry, Vol. 18, No. 4 (July-August 1996), pp. 147-148

### Regrouping, reforming and re-engineering: applying robotics to new challenges

**Stephen Scypinski, Theodore Sadlowski and John Baiano**

*Analytical Research and Development, Hoffmann-La Roche Inc., Nutley, NJ 07110, USA*

*This paper describes the effects of re-engineering, or business process engineering, on the pharmaceutical industry. It then extrapolates the re-engineering fever to the field of analytical chemistry and shows that the use of automation will concomitantly increase over the next several years. This will require a fresh look at projects and situations that can be automated, as well as those thought previously non-automatable. The ability of laboratory personnel to 'think out of the box' will dictate their rise or fall as successful analysts.*

### Business process re-engineering

At the International Symposium on Laboratory Automation and Robotics (ISLAR) over the past several years, many presentations during the plenary and management sessions have dealt with the concept and reality of 'doing much more with much less... faster!' The various forces at work shaping the future of the pharmaceutical industry have affected everyone working in research and development. The containment of research and development costs, coupled with the need to improve profitability without raising prices, have forced drug manufacturers to reorganize, merge, acquire other companies (both large and small), downsize, redirect research efforts, and improve productivity. Intense analyses and benchmarking of companies against one another have made consultants rich and have resulted in the largest wave of change to sweep the pharmaceutical industry since the advent of Federal Drug Laws. Activities which were 'nice to have' are now a thing of the past. Research areas which do not directly result in innovative new products that satisfy medical needs have, or are being, abandoned. Research and development are now driven by the market and the bottom line of the corporation. In today's aggressive competitive situation, companies which do not adopt this strategy will soon be out of the running.

Any new change brings with it a new language. Business analysts have, for several years, preached about the concept of 're-engineering' or, more precisely, 'business process re-engineering'. The concept of business process re-engineering, to quote directly from Hammer and Champy [1], is as follows: 'Business process re-engineering means starting over, starting from scratch. It means putting aside much of the received wisdom of two hundred years of industrial management. It means forgetting how work was done in the age of the mass market and deciding how it can best be done now'.

American business can no longer be content with manufacturing a product that the customer will be 'content' with, or supply a service which an end user will simply 'accept'. The American public now demands the best quality and a product designed and manufactured to their needs and specifications. In order to meet the extreme demands placed upon them, American companies have begun to critically examine the very processes by which they conduct business. The word 'process' is key; many corporations as of now are set up according to *function* and not *process*. This implies that each segment of the business performs a certain task and then passes the work on to the next function. This is very much akin to the concept of assembly-line production made famous by the automobile pioneer Henry Ford in the early years of the 20th century. In the functionality mode, each discrete function performs a given task without regard for what preceded their interaction or what is to follow. This tends to result in products and services which are no more than the sum of their parts; however, the competition has come to deliver products and services which are much more than the sum of their individual functions. It is the latter scenario that forms the basis for business process re-engineering, which considers the process of manufacturing goods or performing a service. In the process mode, a team of individuals who are all thoroughly familiar with the products or service at hand, perform the various tasks, usually in a more efficient manner than the individual functions. How can a smaller team of individuals turn out a better product, faster and more efficiently? The answer is found in the basic question asked during the re-engineering process: 'Do not ask how we can do what we do better, rather ask why we do what we do at all'.

Although this statement casts doubts on the way business is performed now, it is nonetheless true. The concept of functionalization entails having a larger workforce than the process-oriented approach. This is traditionally due to the need for 'doers' and 'checkers' in the functional world, which ultimately results in hierarchical structures. In a process-oriented structure, teams of individuals are empowered by management to make decisions and move a project from injection to completion. These teams of individuals are given the maximum freedom and flexibility within the corporate structure to cause changes in the way the corporation conducts business to occur. Indeed, the formal definition of business process re-engineering summarizes its important concepts:

*Business Process Re-engineering: A set of procedures for effecting radical change in the way a corporation conducts some or all of its business practices: the fundamental rethinking and radical redesign of business processes to achieve dramatic improvements in critical, contemporary measures of performance, such as cost, quality, service and speed.*

### Re-engineering applied to the pharmaceutical industry

The entire concept of business process re-engineering is leaving its mark on the pharmaceutical industry, especially

0142-0453/96 $12.00 © 1996 Taylor & Francis Ltd.

in the research and development area. The demand of the American public on the pharmaceutical industry to control costs has forced companies to react in a multitude of ways. They have merged, acquired other companies, reorganized and downsized. Last year at ISLAR, Dr Philip Lane of R. W. Johnson PRI summarized these trends in a presentation entitled 'The reality of the 90s and beyond—more with less [2]. In his talk, Dr Lane summarized the factors shaping the pharmaceutical industry. These realities include stricter compliance (FDA pre-approval inspections, Barr decision, implementation of ICH guidelines), company mergers and acquisitions, the diversity of the workforce and the necessity of having work and family programmes. For pharmaceutical companies to not only survive but prosper as well, he stated that the ways in which we conduct business must change. This implies a fresh look at old principles with the intention of implementing drastic change.

Since all of the new realities discussed by Dr Lane will impact the bottom line of the corporation, an obvious answer is to either reduce costs and/or improve sales. With the advent of cost restraint, a company's answer to an increased cost of business is no longer raising the prices on the products it markets. It must therefore reduce its cost of doing business, as well as bring new products to market faster and more efficiently. A shorter time in the R&D pipeline means less overall costs which need to be recaptured in profits. Therefore, in theory, products discovered and developed according to a streamlined R&D strategy should be able to be more competitively priced. Indeed, many large research-based pharmaceutical companies are using such techniques as automated high throughput screening assays and combinatorial chemistry to search for 'hits', which may result in a candidate molecule worthy of development. These same corporations are aggressively seeking to improve their track records with respect to bringing new molecules to market faster, while at the same time, not compromising quality and regulatory compliance. 'Breaking down the wall' between research and development is becoming fairly common in the pharmaceutical industry.

### Automation and re-engineering analytical chemistry

It is interesting that Hammer and Champy's text devotes a full chapter to the concept of information technology (IT) as a necessary tool for re-engineering. They stress that many of the re-engineering tasks performed today could not have even been imagined twenty years ago. The power of computing and electronic communication has provided the opportunity to move information instantaneously and without error. This has made the working world considerably smaller and has allowed larger amounts of data and records to be handled by fewer people.

The use of laboratory automation is obviously a part of the concept of re-engineering analytical chemistry. Being hybrids of computers, laboratory robots are a part of the electronic revolution that has swept the industry. Originally introduced into analytical laboratories involved in supporting drug development, robotics techniques are now routinely used in drug discovery as well. In the robotics laboratory in the analytical research and development at Hoffmann-La Roche, the way the business is conducted is being re-engineered. In fact, the creation of the department less than a year ago was a re-engineering step in itself. Since the inception of analytical R&D, the department has constantly strived to look for alternative means of accomplishing its mission while at the same time satisfying the needs of customers and remaining in regulatory compliance.

Last year at ISLAR, the department presented its management philosophy of a centralized/decentralized approach to robotics in which a core group of individuals is charged with the day-to-day operation of the robotics laboratory and its associated equipment [3]. This includes maintenance, troubleshooting, system validation and method development. This core group of robotic specialists then supports the other project-based groups who supply the chemistry and the projects which are transferred to the robots and workstations. The advantages of this hybrid approach are:

(1) There is uniformity in equipment and validation.
(2) There is a mix of expertise in robotics and the chemistry of the compound.
(3) People working on the project benefit from the sharing of ideas and experience.
(4) People working on the automation project can possess ownership without actually having to be a supervisor.

Automation is now being applied to projects in Phases 2 and 3 of clinical trials, as these are the projects which result in the greatest number of samples. The philosophy which was highlighted at last year's ISLAR continues to be in effect. Some of the newest robotic tasks have included the automation of cleaning validation methodology, which is a rather simple but nonetheless mundane task. It is perfectly suited to the Zymark Benchmate. Additional projects now currently underway include automation of animal feed analysis for support of GLP toxicology studies, various biotechnology tests such as bioassay and verification of the exchange of methods with other development centers having the same or similar equipment.

### Conclusions

The use of robotics will play a large part in the re-engineering trends underway in the pharmaceutical industry discovery and development laboratories. The R&D department at Hoffmann-La Roche is a representative cross-section of the industry and its staff are convinced that laboratory automation will truly be one of the concepts that make the 'realities of the 1990s' bear fruit for pharmaceutical companies.

### References

1. Hammer, M. and Champy, J., *Reengineering the Corporation* (1993).
2. Lane, P. A., In *Proceedings of the 1994 ISLAR Meeting* (Zymark, Hopkinton, MA, 1994).
3. Scypinski, S., Nelson, L. and Sadlowski, T., In *Proceedings of the 1994 ISLAR Meeting* (Zymark, Hopkinton, MA, 1994).

